# Leafcutter ants enhance microbial drought resilience in tropical forest soil

**DOI:** 10.1111/1758-2229.13251

**Published:** 2024-05-23

**Authors:** Hannah B. Shulman, Emma L. Aronson, Diego Dierick, Andrian A. Pinto‐Tomás, Jon K. Botthoff, Allan Artavia‐León, Michael F. Allen

**Affiliations:** ^1^ Department of Microbiology and Plant Pathology University of California Riverside California USA; ^2^ Department of Ecology and Evolutionary Biology University of Tennessee Knoxville Tennessee USA; ^3^ Center for Conservation Biology University of California Riverside California USA; ^4^ Department of Biological Sciences Florida International University Miami Florida USA; ^5^ Centro De Investigación En Estructuras Microscópicas Universidad de Costa Rica San José Costa Rica

## Abstract

We conducted a research campaign in a neotropical rainforest in Costa Rica throughout the drought phase of an El‐Nino Southern Oscillation event to determine microbial community dynamics and soil C fluxes. Our study included nests of the leafcutter ant *Atta cephalotes*, as soil disturbances made by these ecosystem engineers may influence microbial drought response. Drought decreased the diversity of microbes and the abundance of core microbiome taxa, including Verrucomicrobial bacteria and Sordariomycete fungi. Despite initial responses of decreasing diversity and altered composition, 6 months post‐drought the microbiomes were similar to pre‐drought conditions, demonstrating the resilience of soil microbial communities to drought events. *A. cephalotes* nests altered fungal composition in the surrounding soil, and reduced both fungal mortality and growth of Acidobacteria post‐drought. Drought increased CH_4_ consumption in soils due to lower soil moisture, and *A. cephalotes* nests decrease the variability of CH_4_ emissions in some soil types. CH_4_ emissions were tracked by the abundance of methanotrophic bacteria and fungal composition. These results characterize the microbiome of tropical soils across both time and space during drought and provide evidence for the importance of leafcutter ant nests in shaping soil microbiomes and enhancing microbial resilience during climatic perturbations.

## Graphical abstract

We carried out an eight‐month investigation on a tropical soil microbial community in the aftermath of a severe drought event, which resulted in changes to soil moisture levels and methane emissions. Our study reveals that the leafcutter ant, *Atta cephalotes*, plays a crucial role in stabilizing the microbial community and enhancing its resilience by mitigating the mortality rates of fungi and methanotrophs. The findings underscore the significance of multi‐trophic interactions in bolstering ecosystem resilience in the face of extreme climatic occurrences.

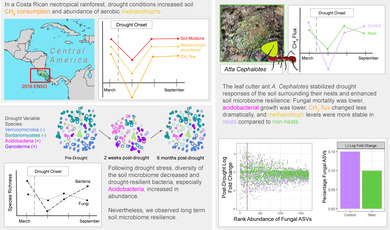

## INTRODUCTION

El Niño/Southern Oscillation (ENSO) events cause heavy rainfall during the La Niña period followed by drought conditions during the El Niño period in much of Central America. In the tropics, the ENSO cycle of 2015–2016 initially increased soil moisture, but then dried out soils and contributed to ecosystem disturbance, including the loss of tropical forest cover, tropical wetlands and aboveground carbon storage (Wigneron et al., 2020; Zhang et al., 2018). Warming climate conditions are projected to increase the frequency of ENSO cycles and drought events, which pose the greatest threat to tropical forest systems (Baldrian et al., 2023; Timmermann et al., 1999). Drought conditions in tropical soils have been shown to alter microbiome phylogenetic patterns and alter C cycling potential (Bouskill et al., 2013; Bouskill et al., 2016; Whitaker et al., 2014). Research into microbial communities under drought in the tropics is needed to mechanistically understand how climatic disturbances alter soil nutrient cycling and carbon (C) cycling.

Leafcutter ants impact the soil microenvironment and C cycling in the tropics: gas escapes through extensively built and well‐maintained ant tunnels and chambers even when surrounding soil is saturated, resulting in 15%–60% higher emission rates of CO_2_ in nests (Fernandez‐Bou et al., 2019). The nests of the leafcutter ant, *Atta cephalotes*, are biogeochemical hotspots of accelerated organic matter turnover and nutrient mineralization due to the rapid decomposition of plant matter by the ant‐cultivated heterotrophic fungi *Leucoagaricus gongylophorus* (Aylward et al., 2013; Fisher et al., 1996; Folgarait, 1998; Swanson et al., 2019). Ant perturbations also have scaling effects on the immediate landscape, ranging from canopy gaps altering air temperature and humidity, decreased litterfall and higher litter turnover, and soil nutrient heterogeneity around nests due to nitrogen leaching (Meyer et al., 2011; Meyer et al., 2013; Verchot et al., 2003). Anthropogenic disturbances in tropical forests are projected to increase the amount of leafcutter ant nests (Siqueira et al., 2017). While the direct physical effects of *Atta* nest architecture on soil C fluxes have been studied in detail, this study aims to determine if the biogeochemical effects of *Atta* nests may indirectly impact fluxes through the soil microbial community.

The objective of this study was to examine the effect of *A. cephalotes* nests on soil fungal and bacterial communities during the drought period of the 2015–2016 ENSO cycle, and determine if the soil microbiome impacted C emissions. In our earlier analysis of methane (CH_4_) fluxes during this ENSO cycle at the neotropical rainforest La Selva Biological Station (Aronson et al., 2019), we observed that CH_4_ consumption increased during the El Niño phase. Soil microorganisms are key producers and consumers of CH_4_. Phylogenetically restricted groups of methanotrophs (Hanson & Hanson, 1996) and methanogens (Garcia et al., 2000) are responsible for the consumption and production of CH_4_, respectively. Environmental changes that impact CH_4_ flux do so by impacting the diversity, composition or balance of these two groups (Aronson et al., 2013). Therefore, we tracked the abundance of methanotrophic bacteria to add to our understanding of how drought can affect CH_4_ dynamics through the microbial community.

Our study addressed the following hypotheses: (1) The drought onset will strongly influence microbial diversity and composition, creating a selection event that results in lower microbial diversity and specific taxonomic shifts. (2) Drier soils with higher CH_4_ consumption will also have less diverse fungal and bacterial communities and a higher abundance of methanotrophs. (3) Ant nests will impact fungal and bacterial composition by increasing diversity, altering the composition and conferring resiliency to soil microbes during the drought period.

## EXPERIMENTAL PROCEDURES

### Study site

This study was conducted at La Selva Biological Station, an old‐growth tropical wet forest reserve in Cordillera Central, Costa Rica. We have previously described climatic and methane flux patterns at this site (Aronson et al., 2019). The mean monthly rainfall at La Selva is typically above 300 mm from May to December with precipitation peaking above 400 mm/month in June–August and November–December and with the driest period in February–March receiving above 150 mm (Organization for Tropical Studies). Due to an El Nino event occurring during this study in 2016, soils were exceptionally dry in April and May The dry conditions peaked on May 8th, when the water content was 27% and the water potential approximately −5 MPa, well below the permanent wilting point (−1.5 MPa) of 33% (Sollins et al., 1994).

### Sample collection

In both residual and soil regions, two plots were set up in areas both occupied by *A. cephalotes* ant nests and in non‐nest control soils (Figure S1). Each plot was divided into four even subplots (*n* = 4), and collars were set up near plot corners, one in each of the four subplots. Soil samples were collected from the top 5 cm of each subplot 4 times during the study period: March, May, July and September of 2016. After collecting, soils were stored at −20C. After the final collection in September, DNA was extracted from all soils using the MOBIO PowerLyzer Powersoil kit (MOBIO Laboratories Inc., Carlsbad CA, Catalogue # 12855‐100) according to the manufacturer's instructions.

Soil CH_4_ flux was collected from the collars using a closed‐chamber system described previously (Aronson et al., 2019). Briefly, collars were sealed with PVC caps and left to incubate for 40 min. Chamber air sampled at three time points was analysed on a gas chromatograph (7890B, Agilent Technologies, Santa Clara, CA, USA) to determine CH_4_ flux. Volumetric water content and soil temperature in the top 5 cm were also collected next to each collar following the flux measurement using a ProCheck handheld datalogger with a GS3 sensor (Decagon Devices, Pullman, WA, USA).

### Marker gene amplicon sequencing

To target bacterial communities, the V3–V4 region of the 16S rRNA gene was amplified from soil DNA extracts using the S‐D‐Bact‐0341‐b‐S‐17 (5′‐CCTACGGGNGGCWGCAG‐3′) and S‐D‐Bact‐0785‐a‐A‐21 (5′‐GACTACHVGGGTATCTAATCC‐3′) primer set (Klindworth et al., 2013). DNA was amplified using KAPA HiFi HotStart ReadyMix (Roche Diagnostics, Indianapolis, IN, USA) and 0.2 μM of each primer. The reaction was carried out with the following thermocycle: Initial denaturing at 95°C for 3 min, followed by 25 cycles of 95°C for 30 s, 55°C for 30 s, 72°C for 30 s and concluding with a final extension at 72°C for 5 min.

To target fungal communities, the internal transcribed spacer 2 (ITS2) region was amplified from soil DNA extracts using the 5.8S‐F (5′‐AACTTTYRRCAAYGGATCWCT‐3′)/ITS4‐FunR (5′‐AGCCTCCGCTTATTGATATGCTTAART‐3′) primer set (Taylor et al., 2016). DNA was amplified using the Phusion High Fidelity Master Mix (NEB), an additional 3 mM MgCl_2_, and 0.2 μM of each primer. The reaction was carried out with the following thermocycle: Initial denaturing at 95°C for 2 min, followed by 35 cycles of 95°C for 30 s, 55°C for 30 s and 60°C for 4 min. Libraries were indexed and then sequenced with paired‐ended 300 bp reads on the Illumina MiSeq platform. The 16S library was sequenced across two MiSeq runs and the ITS2 library was sequenced in a single MiSeq run. These sequence data have been submitted to the SRA under accession number PRJNA749330.

### Bioinformatics

Demultiplexed 16S sequences were analysed in QIIME2 (Bolyen et al., 2019). To minimize batch effect due to sequencing runs, only forward reads were used to analyse bacterial communities. Reads were trimmed to 230 base pairs in QIIME2. DADA2 was then used to remove chimeras, quality filters and sort reads into amplicon sequence variants (ASVs). 16S sequences were assigned taxonomy with a Bayesian classifier using reference sequences from the SILVA database release 132 (Quast et al., 2013). ITS2 reads were processed using the amptk bioinformatics pipeline (Palmer et al., 2018). Briefly, USEARCH9 was used to merge paired‐end reads, cluster sequences and pick ASVs. ASVs were assigned taxonomy using the amptk custom ITS2 database and hybrid taxonomy assignment algorithm. For both 16S and ITS2 datasets, data from a negative sequencing control was used to remove <20 contaminant ASVs. Due to low read counts or rarefactions curves indicating poor library amplification, six bacterial samples and five fungal samples were removed. After processing and filtering, there were a total of 6,363,877 bacterial and 3,622,525 fungal reads classified into 19,142 bacterial and 13,206 fungal ASVs. After filtering out all ASVs whose cumulative read depth accounted for less than 0.05%, there were 1698 bacterial ASVs and 1709 fungal ASVs.

### Microbial community analysis

Community composition was analysed by Principal Coordinate Analysis of an Aitchison distance matrix using the Phyloseq package in R (McMurdie & Holmes, 2013) using ASV counts normalized using centered log‐ratio (clr) transformation in the R package ANCOMBC (Lin & Peddada, 2020). Log fold changes of common ASVs from March to May were calculated using ANCOMBC. Alpha diversity was analysed by calculating species richness, evenness and the ratio of common to rare species richness.

### Methanotroph gene abundance

To determine the abundance of methanotrophic bacteria, we performed quantitative PCR on the *pmoA* 1 gene. Reactions were performed in triplicate using the Biorad C1000 thermocycler. The *pmoA* gene was amplified with the A189F (5′‐GGNGACTGGGACTTCTGG‐3′)/Mb661R (5′‐CCGGMGCAACGTCYTTACC‐3′) primer set (Kolb et al., 2003) at a final concentration of 0.25 μM, 1 μL template and 1× Forget‐Me‐Not EvaGreen qPCR master mix. The final PCR mixture also included 5% PEG, 2.5 mM MgCl_2_ and 250 μg/μL BSA. The PCR reaction was first heated to 37°C for 20 min to denature contaminants and then initial template denaturation was performed at 95°C for 2 min. This was followed by 40 cycles of 95°C for 30 s, 57°C for 45 s, 72°C for 30 s and a final 59°C capture step. The capture step temperature was determined by testing the PCR reaction with a 50–95°C melt curve and used to exclude nonspecific amplification from quantification. A standard curve using the 759 bp *Methylocystis pmoA* BN69_2927 gene was used to calculate the total copies of *pmoA* and standardized to soil weight and moisture. The efficiency of qPCR reactions was at least 84% with *R* ^2^ = 0.999.

### Statistical analysis

Three‐way repeated measures ANOVA models were used to determine the impact of the three experimental factors (soil type, ant nests and sampling month) on soil moisture, CH_4_ fluxes, alpha diversity, the ratio of common:rare taxa richness and microbial gene abundance and expression data. Post hoc analyses included two‐way repeated measure ANOVAs to analyse the impacts of nests and sampling month within each soil group and Tukey tests to compare month‐to‐month means within each soil and nest group.

PERMANOVAs were performed on the Aitchison distance matrices to determine the impact of these 3 experimental factors on microbial composition, as well as the relationship between microbial composition and CH_4_ flux. ANOVAs were run in base R and PERMANOVAs were run using the adonis package. Batch effect was included as a random effect in the analyses of bacterial sequencing data.

## RESULTS AND DISCUSSION

### Microbial communities respond strongly to drought but are resilient long‐term

**FIGURE 1:**
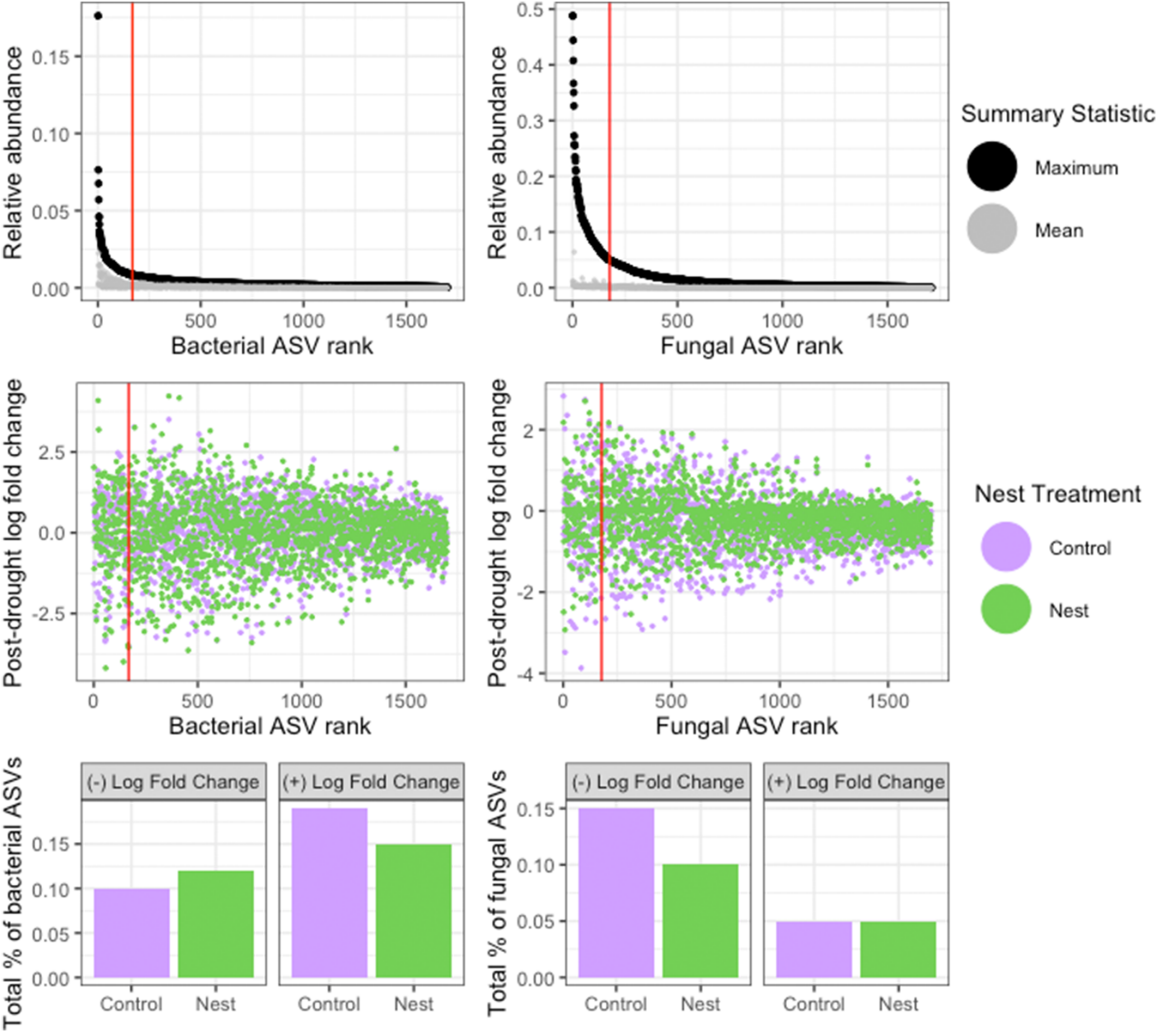
Distribution of microbial abundances and drought responses: bacterial (left column) and fungal (right column) ASVs were ranked across all samples to determine the distribution and drought response of common and rare taxa. The red line indicates the cutoff between the top ~10% of common taxa and the bottom 90% of rare taxa. The *top panel* shows each ASV ranked by maximum relative abundance and the corresponding mean relative abundance from all time points. The *middle panel* shows the log fold change of each ranked ASV from pre‐drought to immediately post‐drought (March–May), the colour represents each ASVs response in control or ant nest plots. The *bottom panel* summarizes the percent of ASVs found to be significantly differentially abundant post‐drought.

To best understand how the microbiome shifted across time, we analysed the diversity and composition of the total microbiome, the ‘common’ microbiome which we defined as the top 10% most abundant taxa, and the ‘rare’ microbiome that makes up the bottom 90%. We chose this cutoff based on the distribution of mean and maximum observed relative abundances for each ASV in our data (Figure 1).

**FIGURE 2:**
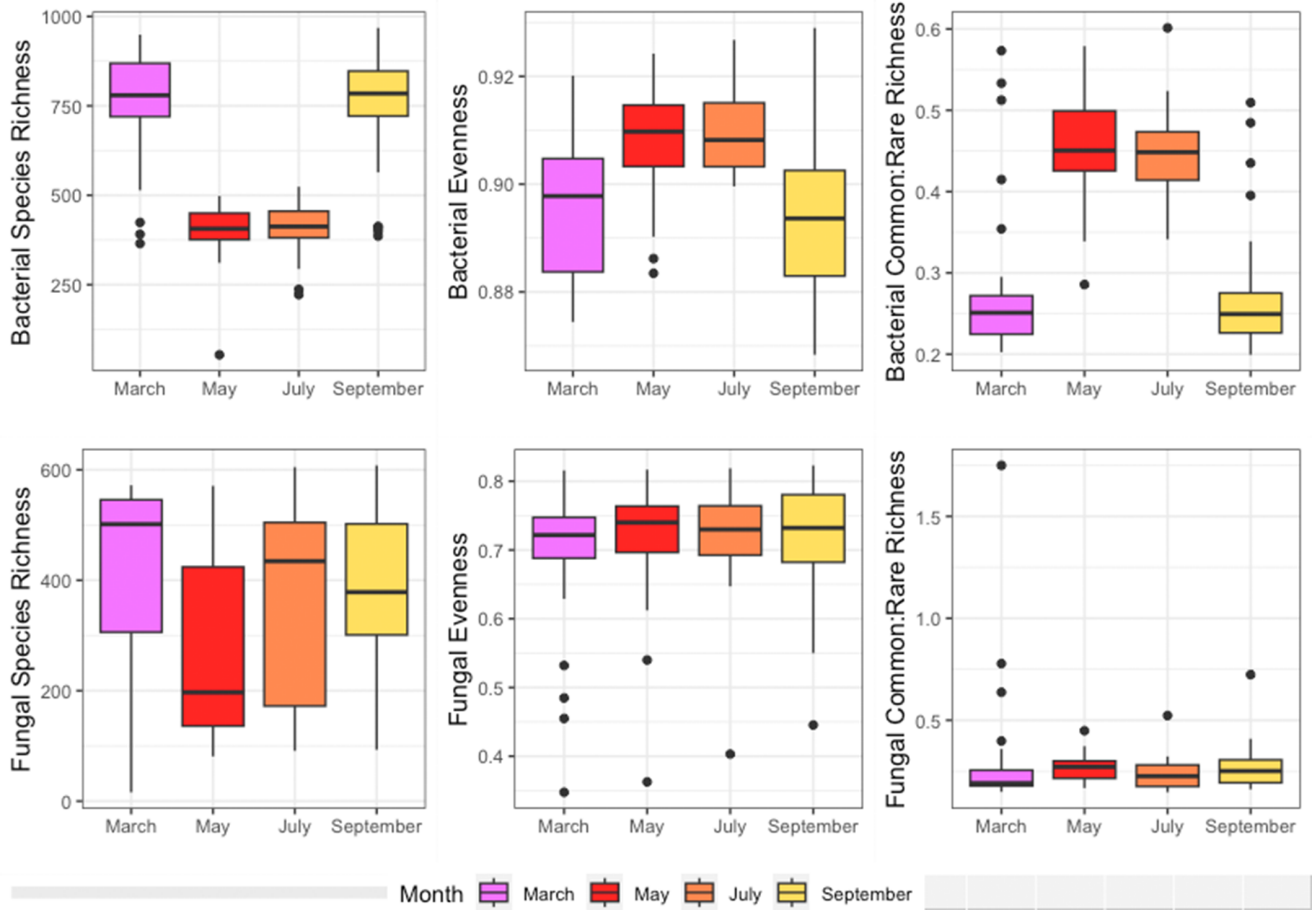
Temporal patterns of microbial diversity: microbial alpha diversity analysed by calculating species richness, evenness and the ratio of common:rare taxa richness for both bacterial and fungal ASVs. The sampling month is indicated by colour. Lower and upper boxplot hinges indicate the 25th and 75th percentiles and whiskers indicate points laying within 1.5 × the IQR.

There was a strong compositional response of total bacteria to the drought event in May (Figure S2, Table S1) characterized by significantly decreasing species richness [*F*(3,28) = 68.301, *p* = 2.5e − 9] (Figure 2). Differential abundance analysis performed with ANCOM (Lin & Peddada, 2020) showed that post drought, composition shifts were driven by 27 decreasing and 70 increasing common bacterial ASVs (Table S3). Approximately half of the decreasing ASVs were from the Verrucomicrobia class Spartobacteria, indicating this group may be especially susceptible to drought stress in the tropics. Spartobacteria are the most abundant Verrucmicrobia in many soil types, and carry out degradation/mineralization of labile organic compounds (Dash et al., 2020), suggesting drought may impact the availability of nutrients indirectly through effects on the soil microbiome. Despite these observed bacterial losses, 15%–18% of bacteria increased in abundance post‐drought (Figure 1), evenness increased [*M* = 0.018, *p* = 0.0009] and the common:rare richness ratio increased. These data show that the common microbiome becomes even more abundant, while rare taxa die back under drought conditions. Our results support the observation that the common or ‘core’ bacterial microbiome is the most resilient to disturbance, and raise questions about the effects of rare taxa death on the biodiversity‐stability relationship of tropical soil microbiomes (De Boeck et al., 2018; Jiao et al., 2019).

Fungi were not as variable over time compared to bacteria (Table S1). Following the drought in May, some fungal communities underwent a compositional shift characterized by decreased species richness (Figure 2, Figure S2), but all other diversity metrics were not variable across time. Differential abundance analysis showed that post‐drought compositional shifts were driven by 29 ASVs decreasing and 17 ASVs increasing (Table S4) in the core fungal microbiome. Several notable saprotrophic species greatly increased post drought including *Tricholoma matsutake*, *Ganoderma australe* and *Ganoderma multiplicatum*. More than half of the decreasing fungal ASVs belonged to the class Sordariomycetes, indicating these taxa may be vulnerable to drought stress in the tropics. These results also contrast with fungal community dynamics in arid systems, where diversity increases with drought due to decreased competition (Hawkes et al., 2011).

Overall, we found support for Hypothesis 1: drought impacted the composition of both fungi and bacteria and reduced alpha diversity, especially for bacteria. The impact of the drought on composition also persisted into July likely because, as previously described in (Aronson et al., 2019), this was when soil saturation and respiration stabilized back to pre‐drought levels. Despite the compositional responses to drought, bacterial and fungal composition and diversity were similar to pre‐drought levels by September (Figure 2). These data suggest that not only are microbial communities resilient long term to acute drought stress, but that the shift seen in bacterial following the drought period was more pronounced than what would be expected from temporal turnover rates in non‐drought conditions, such as was seen in Kivlin and Hawkes (2020), where turnover was consistent over time due to reciprocal gains and losses of taxa.

### Leafcutter ant nests influence microbial drought response

Ants create compartments for fungal gardens in their chambers, cultivating various species of mutualistic Agaricaceae fungi. At La Selva, *A. cephalotes* cultivates the species *Leucoagaricus gongylophorus* (Aylward et al., 2013; Fisher et al., 1996). We found no evidence of this species present in soils around the nest chambers (Figure S3). Although it is likely that fungal gardens containing the fungal symbiont were less than a meter away from our soil cores, ant‐associated *Leucoagaricus* are noncompetitive and rely on ant cultivation for survival (Fisher et al., 1996), which may explain why they do not colonize soil compartments outside the nest.

Nests influenced fungal composition before drought onset (Table S1) but did not change fungal species richness (Table S2a), indicating that the ant‐built environment creates a horizontal shift in fungal taxa, rather than filtering taxa or increasing net niches as seen in (Delgado‐Baquerizo et al., 2019). Furthermore, our results suggest that fungal communities surrounding ant nests may be more resilient to drought, as fewer total nest‐associated ASVs decreased in abundance post‐drought (Figure 1). Amongst the core microbiome there were 17 ASVS which dropped less in abundance in the nests compared to controls (Table S4), notably from the Sordariomycetes order, including *Gliocephalotrichum humicola* and *Gliocladiopsis curvata*.

While we hypothesized that *Atta* nests would select for a specific bacterial community in the soil surrounding their nests, nests had no observed impact on overall bacterial composition (Table S1). The impact of nests on the higher‐level organization of the soil bacterial community may then be confined to the nest compartments (Lucas et al., 2017). However, the nests did impact bacterial drought response, decreasing the percentage of bacteria that increased post‐drought (Figure 1) and specifically decreasing the growth and survival of Acidobacteria (Table S3). As tropical soil microbial response to climate warming and drought is characterized by the dominance of acidobacteria over fungi (Baldrian et al., 2023), our results suggest that leafcutter ants may attenuate the impact of climatic disturbance on soil microbes by enhancing fungal resilience and bacterial resistance to drought stress.

### Leafcutter ant nests alter methane dynamics under drought conditions

**FIGURE 3:**
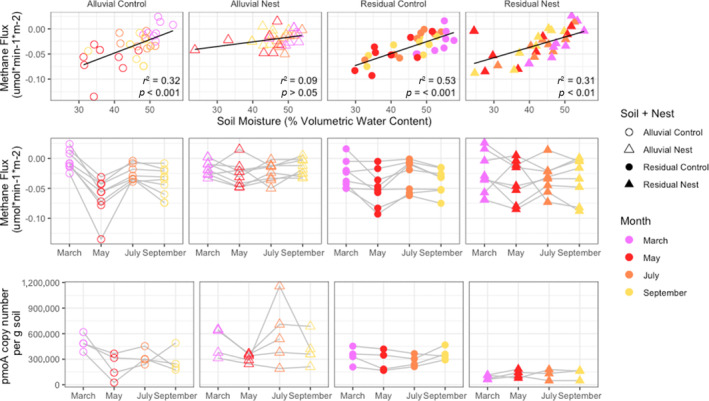
Impact of drought on soil moisture and CH_4_ flux: drought effects on CH_4_ emissions were determined by measuring soil moisture, CH_4_ flux and the abundance of methanotrophic bacteria with qPCR. The *top panel* shows a correlation between soil moisture and CH_4_ flux. Negative flux values indicate soil CH_4_ consumption and positive values indicate CH_4_ production. The sampling month is indicated by colour and ant nests by shape. Trendlines illustrate linear regression between CH_4_ flux and water content, with statistics shown. The *middle panel* of plots shows month‐to‐month CH_4_ flux rates for each collar of the closed chamber system. Individual collars are connected across time points with grey lines. *The bottom panel* of plots shows methanotroph abundance detected by quantifying the *pmoA* gene with qPCR of genomic DNA.

As previously reported (Aronson et al., 2019), in most soils and sampling months these soils acted as a CH_4_ sink, with higher consumption in drier soils (Figure 3). As we hypothesized, the drier soil conditions during drought caused even more CH_4_ consumption (Figure 3). In the alluvial soils, *Atta* decreased the amount of CH_4_ consumed post‐drought, [Control: *M* = −0.0613, *p* = 0.001; Nest: *M* = 0.0094, *p* = 0.997]. This effect is likely because, in nest soils, soil moisture dropped much less significantly during the drought period (Figure 3A), supporting our hypothesis that nests would mitigate the effects of drought.

Methanotroph abundance was detected with quantitative PCR of the *pmoA* gene. *pmoA* encodes the enzyme that catalyses the first step of microbial methanotrophy, methane monooxygenase. Gene copies of *pmoA* ranged from approximately 25,000 to 700,000 copies per gram of soil (Figure 3). The relationship between methanotroph abundance and soil CH_4_ emissions contradicted our hypothesis: post‐drought, there was more consumption of methane, but fewer methanotrophs. It is possible then, that methanotrophs are more abundant in wetter soils when anaerobic methanogens produce more CH_4_, but more efficient at methanotrophy in drier soils. Observed impacts of reduced soil moisture on methanotroph activity vary, with evidence supporting both stimulation (Ma & Lu, 2011) and reduction (Ma et al., 2013) depending on drainage severity. Lastly, in the nests, methanotroph abundance decreased less post‐drought [Control: *M* = −1.7e5, *p* = 0.03; Nest: *M* = −7.02e4, *p* = 0.93] (Figure 3), reiterating the stabilizing effect of the ant nests.

To summarize, we found that *Atta* nests reduce post‐drought fluctuations in CH_4_ consumption, soil moisture and methanotroph abundance. They also reduced fungal mortality and the growth of actinobacteria post‐drought. We propound that these nest effects are due to the rigorous upkeep of nest architecture by *A. cephalotes* and the nutrients added to the soil from leaf inputs, which may buffer the soil microbiome from drought stress (Corrêa et al., 2009; Fernandez‐Bou et al., 2019; Moutinho et al., 2003; Verchot et al., 2003). Furthermore, we found that CH_4_ flux rates were correlated to fungal, but not bacterial, community composition at all time points (Table S1). These data suggest that the influence of *Atta* nests on carbon flux may be through modulation of a complex bacterial‐fungal web of organic matter degradation into CH_4_ and CH_4_ precursors (Blaut, 1994) in addition to their well‐documented physical effects (Fernandez‐Bou et al., 2019; Fernandez‐Bou et al., 2020). Additionally, the effects of bacteria on net CH_4_ may be limited to a small subset of the bacterial community, including the methanotrophs. More information is needed about the C‐cycling capabilities of the uncultured and undescribed bacteria in these soils to determine their relationship to CH_4_ dynamics at the ecosystem function level.

As previously mentioned, this study was performed in both alluvial and residual soils. These soil types impacted baseline microbial community composition, drought response and nest effects on drought and microbial dynamics. The relationship between drought, C fluxes and the soil microbiome were all influenced by soil type, with more methanotrophs and greater nest effects seen in alluvial soil compared to residual. In addition to differences in soil properties, alluvial and residual soils have well‐characterized, distinct plant communities (Clark et al., 1999), which are likely exerting top‐down control of belowground microbial communities (Barberán et al., 2015; Kivlin & Hawkes, 2016). This observed variation across soil types indicates a degree of context dependency that should be considered in future research on microbiome dynamics across the tropics and subtropics. Our experiment demonstrates a case of soil fauna positively impacting microbial resilience during drought, but future studies could investigate if this is seen for other extreme climatic events if these relationships are phylogenetically conserved, and if the biogeochemical capabilities of these microbes may allow for more explicit connection of soil fauna to C flux.

## CONCLUSIONS

In conclusion, we show here that soil microbes respond strongly to drought in the tropics, and that leafcutter ant nests interact with the soil microbial community in complex ways that promote resilience. Future investigations could focus on how nest effects on bacteria and fungi alter the decomposition of organic matter to connect the well‐described soil nutrient and chemical profile of the ant‐built environment (Meyer et al., 2013; Swanson et al., 2019) to community composition and function. Increasing occurrences of drought events in the tropics impact carbon budgets and exacerbate the loss of tropical forests. Understanding how soil microbes respond to climate perturbations is crucial to managing and conserving forest lands and mitigating climate impacts. Our study demonstrates the interconnected way ants, fungi and bacteria respond to a climate disturbance, and points to the importance of understanding interactions between microbes and soil fauna to determine survival and vigour of the soil community in the face of extreme climatic events.

## AUTHOR CONTRIBUTIONS

**Hannah B. Shulman:** Conceptualization (equal); data curation (lead); formal analysis (lead); investigation (lead); methodology (lead); project administration (lead); visualization (lead); writing – original draft (lead); writing – review and editing (lead). **Emma Aronson:** Conceptualization (lead); funding acquisition (lead); investigation (lead); methodology (equal); validation (equal); writing – original draft (equal); writing – review and editing (supporting). **Diego Dierick:** Data curation (equal); formal analysis (equal); investigation (equal); writing – original draft (equal); writing – review and editing (equal). **Andrian A. Pinto‐Tomás:** Conceptualization (equal); investigation (equal); methodology (equal); resources (equal); supervision (equal). **Jon K. Botthoff:** Investigation (equal); methodology (equal). **Allan Artavia‐León:** Investigation (equal); methodology (equal). **Michael Allen:** Conceptualization (lead); funding acquisition (equal); resources (equal); writing – original draft (equal).

## CONFLICT OF INTEREST STATEMENT

The authors declare that they do not have any conflict of interest.

## Supporting information


**Figure S1:** Diagram of Sampling Plot Setup: In two distinct soil types (indicated by coloured fields), we established four plots (indicated by white squares): two containing *Atta cephalotes* ant nests and two non‐nest controls. In each plot, 4 × 20 cm collars (indicated by numbered blue circles) were set up. Soils were sampled from the top 5 cm of each collar in March, May, July and September 2016.
**Figure S2:** Principal component analysis of microbial communities. PCA performed on the Aitchison distance matrix of bacterial and fungal ASVs. Percent variation indicated on each axis. Ellipses represent 95% confidence intervals. The sampling month is represented by colour, soil type by fill and ant nests by shape. PCA of bacterial and fungal communities explained 35% and 30% of variation in the first three axes, respectively.
**Table S1:** PERMANOVA results analysing the drivers of bacterial and fungal community composition. Tests were run on an Aitchison distance matrix. (A)The effect of soil type, sampling month and ant nests. (B) The correlation between microbial community composition and CH_4_ fluxes over time.
**Figure S3:** Soil around ant nests does not contain *Atta* associated species: The average relative abundance of total Agaricaceae fungi for each combination of nest and soil type (left) as well as the proportion of different species (right) within each sample.
**Table S2:** ANOVA results analysing the effect of sampling month, soil type and ant nests on metrics of fungal and bacterial alpha diversity.
**Table S3:** The 97 bacterial ASVs that were differentially abundant post‐drought. Calculated by analysing the most abundant 10% of ASVs with ANCOM‐BC. ASVs are grouped based on taxonomic assignments. Red taxa (27) are less abundant post‐drought, and blue taxa (70) are more abundant post‐drought. Log fold change (lfc) and p‐values are shown for both nest and control plots.
**Table S4:** The 46 fungal ASVs that were differentially abundant post‐drought. Calculated by analysing the most abundant 10% of ASVs with ANCOM‐BC. ASVs are grouped based on taxonomic assignments. Red taxa (29) are less abundant post‐drought, and blue taxa (17) are more abundant post‐drought. Log fold change (lfc) and *p*‐values are shown for both nest and control plots.

**Data S1:** Supporting Information.

## Data Availability

The sequence data that support the findings of this study are openly available in the NCBI SRA at https://www.ncbi.nlm.nih.gov/bioproject/PRJNA749330 reference number PRJNA749330. Other data are available from the corresponding author, HS, upon reasonable request.

## Footnotes

^1^
To investigate methanotroph abundance in this study, we used a primer set that targets the largest available methanotroph diversity (Kolb et al., 2003), capturing both gammaproteobacterial type I methanotrophs and alphaproteobacterial type II methanotrophs (Hanson & Hanson, 1996). However, this primer set has been demonstrated to have limited efficacy capturing the diversity of Verrucomicrobial methanotrophs (Ghashghavi et al., 2017), a group that was also detected in our 16S survey data (specifically, ASVs belonging to the genus *Methylacidiphulum*). Based on this research campaign, we would recommend that investigation into methanotrophy at La Selva or similar tropical forest ecosystems take into account Verrucomicrobial methanotrophs, which may be more widespread outside extremophilic niches than originally thought (van Teeseling et al., 2014) and are capable of carrying out anaerobic methane oxidizing reactions (Op den Camp et al., 2009).
