# Supplementary material for: Leafcutter ants enhance microbial drought resilience in tropical forest soil
Source: Environ Microbiol Rep. 2024 May 23;16(3):e13251. doi: 10.1111/1758-2229.13251 (PMC11112399; doi:10.1111/1758-2229.13251)
Supplement: Supplementary file 1 — Figure S1: Diagram of Sampling Plot Setup: In two distinct soil types (indicated by coloured fields), we established four plots (indicated by white squares): two containing Atta cephalotes ant nests and two non‐nest controls. In each plot, 4 × 20 cm collars (indicated by numbered blue circles) were set up. Soils were sampled from the top 5 cm of each collar in March, May, July and September 2016. Figure S2: Principal component analysis of microbial communities. PCA performed on the Aitchison distance matrix of bacterial and fungal ASVs. Percent variation indicated on each axis. Ellipses represent 95% confidence intervals. The sampling month is represented by colour, soil type by fill and ant nests by shape. PCA of bacterial and fungal communities explained 35% and 30% of variation in the first three axes, respectively. Table S1: PERMANOVA results analysing the drivers of bacterial and fungal community composition. Tests were run on an Aitchison distance matrix. (A)The effect of soil type, sampling month and ant nests. (B) The correlation between microbial community composition and CH4 fluxes over time. Figure S3: Soil around ant nests does not contain Atta associated species: The average relative abundance of total Agaricaceae fungi for each combination of nest and soil type (left) as well as the proportion of different species (right) within each sample. Table S2: ANOVA results analysing the effect of sampling month, soil type and ant nests on metrics of fungal and bacterial alpha diversity. Table S3: The 97 bacterial ASVs that were differentially abundant post‐drought. Calculated by analysing the most abundant 10% of ASVs with ANCOM‐BC. ASVs are grouped based on taxonomic assignments. Red taxa (27) are less abundant post‐drought, and blue taxa (70) are more abundant post‐drought. Log fold change (lfc) and p‐values are shown for both nest and control plots. Table S4: The 46 fungal ASVs that were differentially abundant post‐drought. Calculated by analysing the most [file EMI4-16-e13251-s001.pdf]

Supplementary Figure 1: Diagram of Sampling Plot Setup: In two distinct soil types (indicated by colored fields), we established 4 plots (indicated by white squares): two containing *Atta cephalotes* ant nests and two non-nest controls. In each plot, 4 x 20cm collars (indicated by numbered blue circles) were set up. Soils were sampled from the top 5cm of each collar in March, May, July, and September 2016.

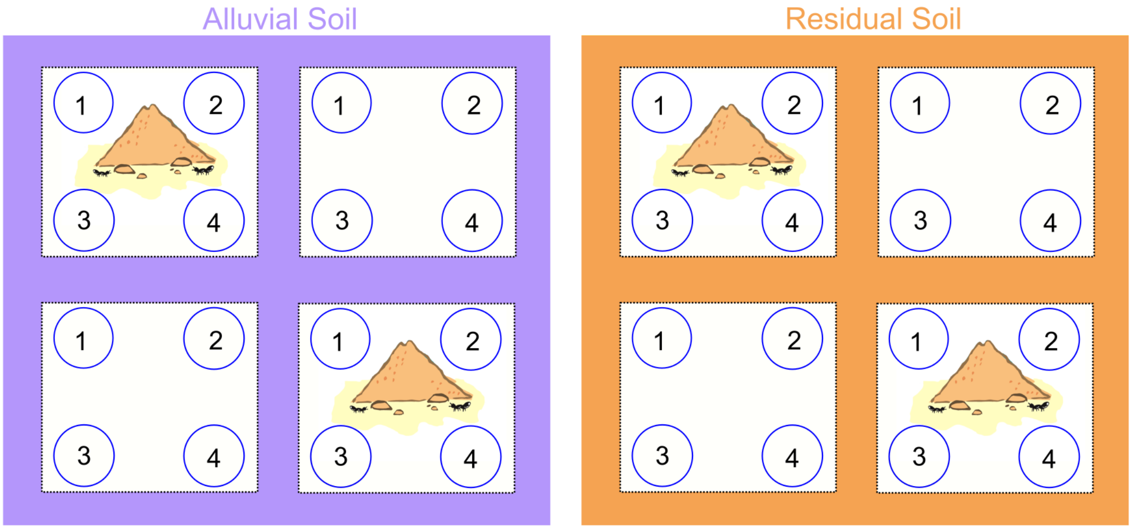

<u>Supplementary Figure 2: Principal Component Analysis of Microbial Communities.</u> PCA performed on aitchison distance matrix of bacterial and fungal ASVs. Percent variation indicated on each axis. Ellipses represent 95% confidence intervals. Sampling month represented by color, soil type by fill, and ant nests by shape. PCA of bacterial and fungal communities explained 35% and 30% of variation in the first 3 axes, respectively.

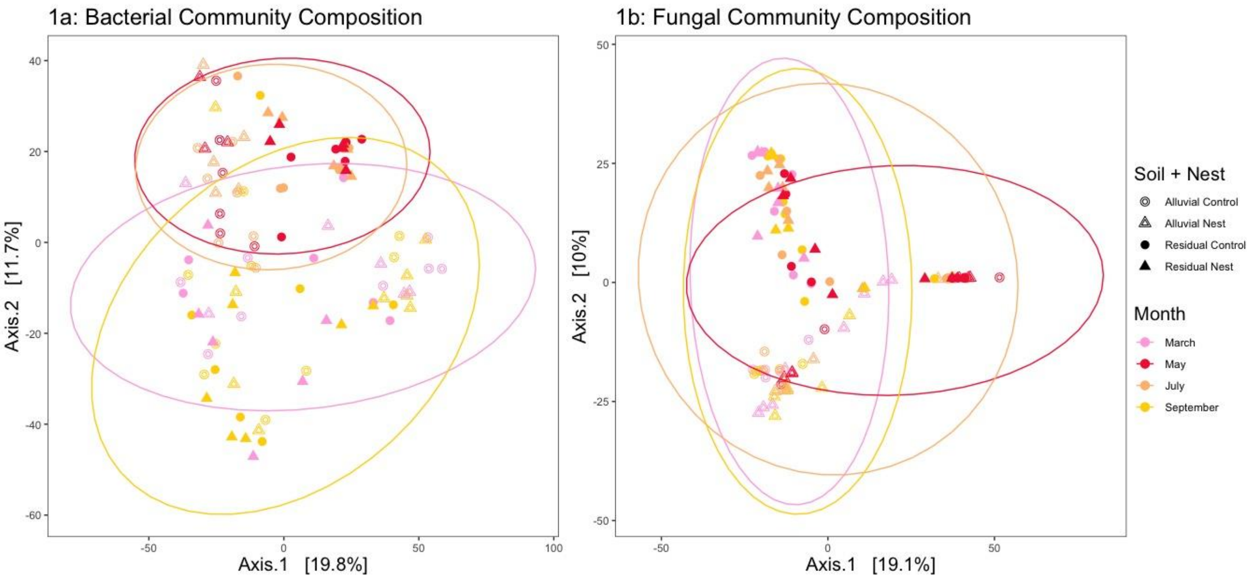

Supplementary Table 1: PERMANOVA results analyzing the drivers of bacterial and fungal community composition. Test were run on an aitchison distance matrix. A)The effect of soil type, sampling month, and ant nests. B) The correlation between microbial community composition and CH_4 fluxes over time

**Table A:**	**Bacteria**					**Fungi**				
**Factor**	**Df**	**SumSq**	**F**	**R^2**	**P**	**Df**	**SumSq**	**F**	**R^2**	**P**
Soil	1	8357	1.907	0.017	0.258	1	20754`	6.880	0.063	.034
Month	3	53689	4.0827	0.108	0.049	3	20477	2.200	0.062	.002
AntNest	1	4244	0.968	0.009	0.258	1	5767	1.859	0.018	.033
Soil:Month	3	28085	2.136	0.057	0.369	3	8285	0.890	0.025	0.528
Soil:AntNest	1	5820	1.328	0.012	0.063	1	6006	1.936	0.018	0.029
Residuals	85	372599		0.752		82				

**Table B:**	**Bacteria**					**Fungi**				
**Factor**	**Df**	**SumSq**	**F**	**R^2**	**P**	**Df**	**SumSq**	**F**	**R^2**	**P**
Month	3	39056	3.782	0.103	0.002	3	13778	1.785	0.052	0.004
CH_4 flux	1	4750	1.380	0.013	0.128	1	5286	2.055	0.020	0.007
Month:CH_4 flux	3	11176	1.0822	0.030	0.281	3	9484	1.238	0.036	0.110
Residuals	94	323599		0.855		96	247018		0.929	

Supplementary Figure 3: Soil Around Ant Nests Does Not Contain *Atta* Associated Species: The average relative abundance of total Agaricaceae fungi for each combination of nest and soil type (left) as well as the proportion of different species (right) within each sample.

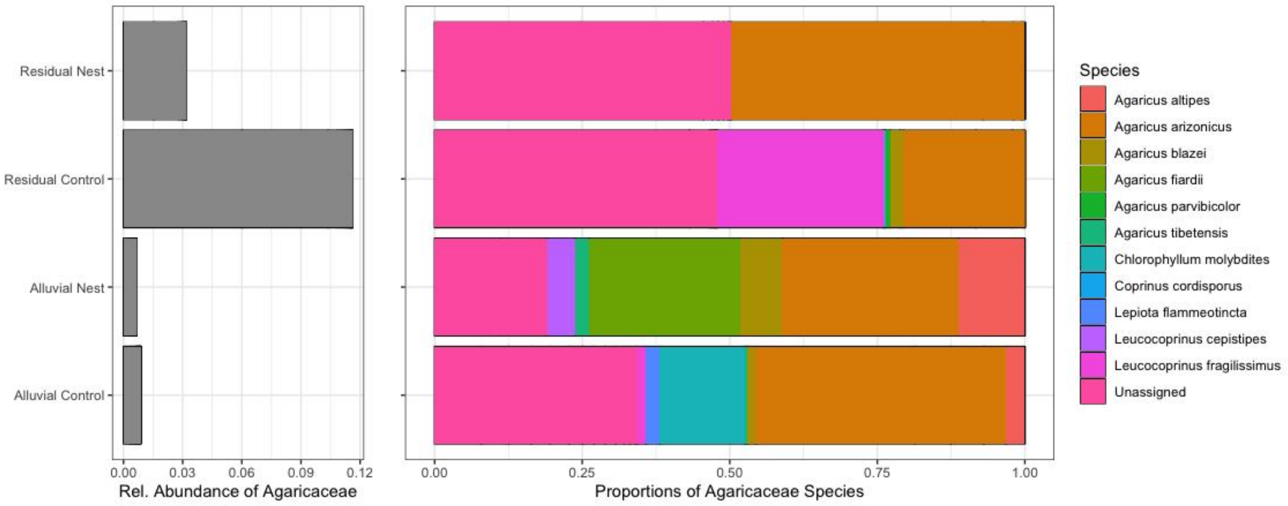

Supplementary Table 2: ANOVA results analyzing the effect of sampling month, soil type, and ant nests on metrics of fungal and bacterial alpha diversity.

	**A: Species Richness**							
	**Bacteria**				**Fungi**			
**Factor**	**Df**	**SumSq**	**FValue**	**P**	**Df**	**SumSq**	**FValue**	**P**
Soil	1	210	0.012	0.915	1	5.4e4	2.023	0.159
AntNest	1	2.4e4	1.176	0.281	1	717	0.027	0.871
Month	3	3.0e6	55.312	2e-6	3	2.8e5	3.455	0.020
Soil:AntNest	1	358	0.020	0.889	1	1.1e5	4.269	0.042
Soil:Month	3	5.1e5	0.919	.0451	3	3.9e4	0.485	0.694
Residuals	86	1.6e6			85	2.3e6		

	**B: Evenness**							
	**Bacteria**				**Fungi**			
**Factor**	**Df**	**SumSq**	**FValue**	**P**	**Df**	**SumSq**	**FValue**	**P**
Soil	1	1.9e-4	1.292	0.259	1	0.011	1.220	0.272
AntNest	1	1.34e-4	0.912	0.342	1	0.045	5.220	0.025
Month	3	5.4e-3	12.230	9.7e-7	3	0.016	0.625	0.600
Soil:AntNest	1	1.9e-5	0.127	0.722	1	0.018	2.066	0.154
Soil:Month	3	6.4e-4	1.444	0.236	3	0.022	0.851	0.469
Residuals	86	0.012			85	0.7391		

	**C: Common:rare species richness**							
	**Bacteria**				**Fungi**			
**Factor**	**Df**	**SumSq**	**FValue**	**P**	**Df**	**SumSq**	**FValue**	**P**
Soil	1	2.9e-3	1.709	0.195	1	0.012	4.374	0.040
AntNest	1	6.4e-3	3.764	0.056	1	0.010	3.443	0.067
Month	3	0.202	39.207	4.6e-9	3	0.014	1.648	0.185
Soil:AntNest	1	0.000	0.000	0.992	1	0.007	2.574	0.112
Soil:Month	3	0.018	3.652	0.016	3	0.027	3.182	0.028
Residuals	86				85	0.2382		

Supplementary Table 3: The 97 bacterial ASVs that were differentially abundant post-drought. Calculated by analyzing the most abundant 10% of ASVs with ANCOM-BC. ASVs are grouped based on taxonomic assignments. Red taxa (27) are less abundant post drought, blue taxa (70) are more abundant post drought. Log fold change (lfc) and p-values are shown for both nest and control plots.

Lfc control	Lfc nest	Pval control	Pval nest	Phylum	Class	Order	Family	Genus	Species
-1.9331	-2.1151	0.0077	0.0221	Acidobacteria	Subgroup 6	Unassigned	Unassigned	Unassigned	Unassigned
-1.8761	-0.5077	0.0000	0.5712	Acidobacteria	Subgroup 6	Unassigned	Unassigned	Unassigned	Unassigned
-1.5866	-1.1133	0.0051	0.2775	Acidobacteria	Subgroup 6	Unassigned	Unassigned	Unassigned	Unassigned
-3.2912	-4.1873	0.0000	0.0000	Actinobacteria	Acidimicrobiia	Acidimicrobiales	uncultured	Unassigned	Unassigned
-2.1349	-2.3178	0.0014	0.0025	Actinobacteria	Acidimicrobiia	Acidimicrobiales	uncultured	Unassigned	Unassigned
-1.5570	0.0683	0.0030	0.9526	Chloroflexi	JG37-AG-4	Unassigned	Unassigned	Unassigned	Unassigned
-3.5167	-3.5559	0.0000	0.0000	Firmicutes	Clostridia	Halanaerobiales	ODP1230B8.23	Unassigned	Unassigned
-1.9538	-3.0649	0.0030	0.0000	Planctomycetes	Planctomycetacia	Planctomycetales	Planctomycetaceae	Planctomyces	Unassigned
-2.3926	-1.2680	0.0001	0.0508	Planctomycetes	Planctomycetacia	Planctomycetales	Planctomycetaceae	Planctomyces	Unassigned
-1.7352	-1.8391	0.0088	0.0016	Planctomycetes	Planctomycetacia	Planctomycetales	Planctomycetaceae	Planctomyces	Unassigned
-1.1600	-1.3286	0.0183	0.1661	Planctomycetes	Planctomycetacia	Planctomycetales	Planctomycetaceae	Planctomyces	Unassigned
-2.2416	-2.0783	0.0001	0.0008	Planctomycetes	Planctomycetacia	Planctomycetales	Planctomycetaceae	uncultured	Unassigned
-1.9992	0.0443	0.0024	0.9671	Proteobacteria	Alphaproteobacteria	Caulobacterales	Caulobacteraceae	uncultured	Unassigned
-0.6800	-0.4468	0.0000	0.1375	Proteobacteria	Alphaproteobacteria	Rhizobiales	Xanthobacteraceae	Variibacter	Unassigned
-1.4053	-2.1047	0.0174	0.0067	Proteobacteria	Alphaproteobacteria	Rhodospirillales	DA111	Unassigned	Unassigned
-0.6093	-2.2577	0.4426	0.0032	Proteobacteria	Betaproteobacteria	SC-I-84	Unassigned	Unassigned	Unassigned
-2.8049	-3.4730	0.0000	0.0000	Verrucomicrobia	Spartobacteria	Chthoniobacterales	DA101 soil group	Unassigned	Unassigned
-2.6108	-3.0096	0.0000	0.0000	Verrucomicrobia	Spartobacteria	Chthoniobacterales	DA101 soil group	Unassigned	Unassigned
-3.3599	-3.1681	0.0000	0.0000	Verrucomicrobia	Spartobacteria	Chthoniobacterales	DA101 soil group	Unassigned	Unassigned
-2.9501	-2.6907	0.0000	0.0003	Verrucomicrobia	Spartobacteria	Chthoniobacterales	DA101 soil group	Unassigned	Unassigned
-2.0529	-0.5948	0.0123	0.5384	Verrucomicrobia	Spartobacteria	Chthoniobacterales	DA101 soil group	Unassigned	Unassigned
-1.8487	-2.2472	0.0006	0.0000	Verrucomicrobia	Spartobacteria	Chthoniobacterales	DA101 soil group	Unassigned	Unassigned

-1.4870	-2.7385	0.0491	0.0001	Verrucomicrobia	Spartobacteria	Chthoniobacterales	DA101 soil group	Unassigned	Unassigned
-1.1083	-2.0748	0.1441	0.0017	Verrucomicrobia	Spartobacteria	Chthoniobacterales	DA101 soil group	Unassigned	Unassigned
-2.9172	-3.9900	0.0001	0.0000	Verrucomicrobia	Spartobacteria	Chthoniobacterales	DA101 soil group	Unassigned	Unassigned
-2.6204	-2.7104	0.0000	0.0000	Verrucomicrobia	Spartobacteria	Chthoniobacterales	DA101 soil group	Unassigned	Unassigned
-0.9387	-2.4320	0.2570	0.0001	Verrucomicrobia	Spartobacteria	Chthoniobacterales	DA101 soil group	Unassigned	Unassigned
0.9654	0.4204	0.0000	0.5044	Acidobacteria	Acidobacteria	Acidobacteriales	Acidobacteriaceae (Sbgrp 1)	Unassigned	Unassigned
1.2414	0.8337	0.0000	0.2209	Acidobacteria	Acidobacteria	Acidobacteriales	Acidobacteriaceae (Sbgrp 1)	Unassigned	Unassigned
0.3000	-0.0074	0.0442	0.9905	Acidobacteria	Acidobacteria	Acidobacteriales	Acidobacteriaceae (Sbgrp 1)	uncultured	Unassigned
0.7241	1.1261	0.0000	0.0000	Acidobacteria	Acidobacteria	Acidobacteriales	Acidobacteriaceae (Sbgrp 1)	uncultured	Unassigned
0.8211	-0.2017	0.0002	0.7347	Acidobacteria	Acidobacteria	Acidobacteriales	Acidobacteriaceae (Sbgrp 1)	uncultured	Unassigned
1.0205	-0.9844	0.0045	0.2498	Acidobacteria	Acidobacteria	Acidobacteriales	Acidobacteriaceae (Sbgrp 1)	uncultured	Unassigned
1.4973	0.0582	0.0487	0.9322	Acidobacteria	Acidobacteria	Acidobacteriales	Acidobacteriaceae (Sbgrp 1)	uncultured	Unassigned
1.5652	0.4904	0.0004	0.5786	Acidobacteria	Acidobacteria	Acidobacteriales	Acidobacteriaceae (Sbgrp 1)	uncultured	Unassigned
0.9257	0.5111	0.0000	0.4309	Acidobacteria	Solibacteres	Solibacterales	Solibacteraceae (Sbgrp 3)	Bryobacter	Unassigned
1.0155	0.5760	0.0000	0.3635	Acidobacteria	Solibacteres	Solibacterales	Solibacteraceae (Sbgrp 3)	Bryobacter	Unassigned
1.1283	0.3975	0.0000	0.6653	Acidobacteria	Solibacteres	Solibacterales	Solibacteraceae (Sbgrp 3)	Bryobacter	Unassigned
0.5213	-0.1907	0.0040	0.7821	Acidobacteria	Solibacteres	Solibacterales	Solibacteraceae (Sbgrp 3)	Candidatus Solibacter	Unassigned
0.8813	0.3380	0.0000	0.6373	Acidobacteria	Solibacteres	Solibacterales	Solibacteraceae (Sbgrp 3)	Candidatus Solibacter	Unassigned
0.8664	0.9348	0.0000	0.0000	Acidobacteria	Solibacteres	Solibacterales	Solibacteraceae (Sbgrp 3)	Candidatus Solibacter	Unassigned
0.8664	1.5600	0.0000	0.0920	Acidobacteria	Subgroup 17	Unassigned	Unassigned	Unassigned	Unassigned
0.7156	0.6481	0.0010	0.3774	Acidobacteria	Subgroup 2	Unassigned	Unassigned	Unassigned	Unassigned
0.3289	-0.3739	0.0226	0.5794	Acidobacteria	Subgroup 2	Unassigned	Unassigned	Unassigned	Unassigned
0.7868	0.2652	0.0000	0.6357	Acidobacteria	Subgroup 2	Unassigned	Unassigned	Unassigned	Unassigned
1.2519	2.2042	0.0042	0.0116	Acidobacteria	Subgroup 2	Unassigned	Unassigned	Unassigned	Unassigned
1.4272	0.9348	0.0084	0.0000	Acidobacteria	Subgroup 2	Unassigned	Unassigned	Unassigned	Unassigned
1.7001	1.9909	0.0159	0.0199	Acidobacteria	Subgroup 2	Unassigned	Unassigned	Unassigned	Unassigned
0.5524	0.1404	0.0000	0.8181	Acidobacteria	Subgroup 5	Unassigned	Unassigned	Unassigned	Unassigned
0.8023	1.3226	0.0176	0.1088	Acidobacteria	Subgroup 6	Unassigned	Unassigned	Unassigned	Unassigned

1.2062	1.3858	0.0005	0.1188	Acidobacteria	Subgroup 6	Unassigned	Unassigned	Unassigned	Unassigned
1.4067	1.0523	0.0000	0.0461	Actinobacteria	Actinobacteria	Corynebacteriales	Mycobacteriaceae	Mycobacterium	Unassigned
1.0741	0.3279	0.0016	0.5003	Actinobacteria	Actinobacteria	Frankiales	Acidothermaceae	Acidothermus	Unassigned
0.7875	0.2527	0.0000	0.6223	Actinobacteria	Actinobacteria	Frankiales	Acidothermaceae	Acidothermus	Unassigned
1.0439	0.7224	0.0000	0.1799	Actinobacteria	Actinobacteria	Frankiales	Acidothermaceae	Acidothermus	Unassigned
1.3364	0.4314	0.0040	0.4340	Actinobacteria	Actinobacteria	Frankiales	Acidothermaceae	Acidothermus	Unassigned
0.8664	1.7001	0.0000	0.0367	Actinobacteria	Actinobacteria	Propionibacteriales	Nocardioidaceae	Unassigned	Unassigned
0.3172	1.0212	0.3918	0.0486	Bacteroidetes	Sphingobacteriia	Sphingobacteriales	Chitinophagaceae	Unassigned	Unassigned
0.8664	1.7101	0.0000	0.0377	Chloroflexi	JG37-AG-4	Unassigned	Unassigned	Unassigned	Unassigned
0.8664	1.7210	0.0000	0.0388	Chloroflexi	JG37-AG-4	Unassigned	Unassigned	Unassigned	Unassigned
0.8664	1.4141	0.0000	0.1102	Chloroflexi	JG37-AG-4	Unassigned	Unassigned	Unassigned	Unassigned
0.8745	-0.2941	0.0000	0.6303	Chloroflexi	Ktedonobacteria	Ktedonobacterales	HSB OF53-F07	Unassigned	Unassigned
1.8321	1.5525	0.0004	0.0017	Chloroflexi	Ktedonobacteria	Ktedonobacterales	Unassigned	Unassigned	Unassigned
1.6404	1.8983	0.0000	0.0231	Chloroflexi	Unassigned	Unassigned	Unassigned	Unassigned	Unassigned
0.8664	1.6589	0.0000	0.0325	Firmicutes	Bacilli	Lactobacillales	Streptococcaceae	Streptococcus	Unassigned
0.7227	-0.8222	0.0004	0.3120	Firmicutes	Clostridia	Halanaerobiales	ODP1230B8.23	Unassigned	Unassigned
1.2388	3.1911	0.0140	0.0007	GAL15	Unassigned	Unassigned	Unassigned	Unassigned	Unassigned
1.9145	4.0928	0.0070	0.0003	GAL15	Unassigned	Unassigned	Unassigned	Unassigned	Unassigned
1.1410	1.6777	0.0001	0.0344	Gemmatimonadetes	Gemmatimonadetes	Gemmatimonadales	Gemmatimonadaceae	uncultured	Unassigned
0.4471	-0.1727	0.0280	0.7839	Nitrospirae	Nitrospira	Nitrospirales	Nitrospiraceae	Nitrospira	Unassigned
1.4368	0.9488	0.0001	0.1891	Planctomycetes	Planctomycetacia	Planctomycetales	Planctomycetaceae	Singulisphaera	Unassigned
1.4577	0.2145	0.0001	0.7873	Planctomycetes	Planctomycetacia	Planctomycetales	Planctomycetaceae	Unassigned	Unassigned
2.4703	1.8738	0.0044	0.0365	Planctomycetes	Planctomycetacia	Planctomycetales	Planctomycetaceae	uncultured	Unassigned
0.8664	1.6578	0.0000	0.0323	Proteobacteria	Alphaproteobacteria	Rhizobiales	Xanthobacteraceae	Unassigned	Unassigned
0.6683	1.4223	0.0012	0.0879	Proteobacteria	Alphaproteobacteria	Rhodospirillales	Acetobacteraceae	Acidiphilium	Unassigned
0.2750	1.3031	0.4495	0.0000	Proteobacteria	Alphaproteobacteria	Rhodospirillales	DA111	Unassigned	Unassigned
0.8468	1.8559	0.0228	0.0386	Proteobacteria	Alphaproteobacteria	Rhodospirillales	DA111	Unassigned	Unassigned
0.8664	1.6777	0.0000	0.0344	Proteobacteria	Alphaproteobacteria	Rhodospirillales	DA111	Unassigned	Unassigned

0.8664	1.6875	0.0000	0.0354	Proteobacteria	Alphaproteobacteria	Rhodospirillales	DA111	Unassigned	Unassigned
0.8664	1.7139	0.0000	0.0381	Proteobacteria	Alphaproteobacteria	Rhodospirillales	DA111	Unassigned	Unassigned
0.8702	0.2402	0.0000	0.6604	Proteobacteria	Alphaproteobacteria	Rhodospirillales	DA111	Unassigned	Unassigned
0.8664	2.0239	0.0000	0.0692	Proteobacteria	Alphaproteobacteria	Rhodospirillales	Rhodospirillaceae	Azospirillum	Unassigned
0.8100	1.3726	0.0380	0.1227	Proteobacteria	Betaproteobacteria	Burkholderiales	Burkholderiaceae	Unassigned	Unassigned
2.3742	0.6840	0.0037	0.4572	Proteobacteria	Betaproteobacteria	Unassigned	Unassigned	Unassigned	Unassigned
0.8664	1.6815	0.0000	0.0348	Proteobacteria	Deltaproteobacteria	Desulfurellales	Desulfurellaceae	H16	Unassigned
1.2478	1.1067	0.0000	0.0694	Proteobacteria	Deltaproteobacteria	Desulfurellales	Desulfurellaceae	H16	Unassigned
0.4061	-0.1899	0.0000	0.7315	Proteobacteria	Deltaproteobacteria	Myxococcales	Polyangiaceae	Sorangium	Unassigned
0.2344	0.9348	0.6173	0.0000	Proteobacteria	Gammaproteobacteria	Enterobacteriales	Enterobacteriaceae	Unassigned	Unassigned
0.8664	1.7431	0.0000	0.0411	Proteobacteria	Gammaproteobacteria	Legionellales	Coxiellaceae	Aquicella	Unassigned
0.8664	1.7523	0.0000	0.0420	Proteobacteria	Gammaproteobacteria	Legionellales	Coxiellaceae	Aquicella	Unassigned
0.8664	1.7850	0.0000	0.0454	Proteobacteria	Gammaproteobacteria	Pseudomonadales	Moraxellaceae	Acinetobacter	Unassigned
0.8664	1.5054	0.0000	0.0057	Unassigned	Unassigned	Unassigned	Unassigned	Unassigned	Unassigned
0.8664	1.6692	0.0000	0.0160	Unassigned	Unassigned	Unassigned	Unassigned	Unassigned	Unassigned
1.3506	0.9348	0.0049	0.0000	Unassigned	Unassigned	Unassigned	Unassigned	Unassigned	Unassigned
1.7969	1.5783	0.0081	0.0097	Unassigned	Unassigned	Unassigned	Unassigned	Unassigned	Unassigned
0.7274	1.6642	0.0000	0.0330	Verrucomicrobia	Spartobacteria	Chthoniobacterales	Chthoniobacterales I.S.	Terrimicrobium	Unassigned
1.2487	0.4062	0.0294	0.6454	Verrucomicrobia	Spartobacteria	Chthoniobacterales	Unassigned	Unassigned	Unassigned

Supplementary Table 4: The 46 fungal ASVs that were differentially abundant post-drought. Calculated by analyzing the most abundant 10% of ASVs with ANCOM-BC. ASVs are grouped based on taxonomic assignments. Red taxa (29) are less abundant post drought, blue taxa (17) are more abundant post drought. Log fold change (lfc) and p-values are shown for both nest and control plots.

lfc.control	lfc.nest	pval.control	pval.nest	Phylum	Class	Order	Family	Genus	Species
-0.6916	0.2534	0.0221	0.6853	Ascomycota	Dothideomycetes	Capnodiales	Cladosporiaceae	Cladosporium	C. halotolerans
-0.5714	-2.4920	0.3727	0.0217	Ascomycota	Dothideomycetes	Pleosporales	Didymellaceae	Unassigned	Unassigned
-1.3031	-0.6213	0.0422	0.1676	Ascomycota	Pezizomycetes	Pezizales	Unassigned	Unassigned	Unassigned
-1.9421	-1.4255	0.0430	0.0758	Ascomycota	Sordariomycetes	Diaporthales	Unassigned	Unassigned	Unassigned
-1.4767	-0.1903	0.0000	0.3704	Ascomycota	Sordariomycetes	Glomerellales	Glomerellaceae	Colletotrichum	C. musae
-2.8869	-2.0754	0.0007	0.0121	Ascomycota	Sordariomycetes	Hypocreales	Hypocreaceae	Trichoderma	T. longipilis
-2.7234	-1.3559	0.0024	0.0504	Ascomycota	Sordariomycetes	Hypocreales	Nectriaceae	Gliocephalotrichum	G. humicola
-2.1467	-1.2166	0.0177	0.1705	Ascomycota	Sordariomycetes	Hypocreales	Nectriaceae	Gliocladiopsis	G. curvata
-1.9381	-2.1952	0.0019	0.0022	Ascomycota	Sordariomycetes	Hypocreales	Unassigned	Unassigned	Unassigned
-1.8143	-1.2730	0.0394	0.1257	Ascomycota	Sordariomycetes	Hypocreales	Unassigned	Unassigned	Unassigned
-1.5222	-0.4158	0.0194	0.5481	Ascomycota	Sordariomycetes	Hypocreales	Unassigned	Unassigned	Unassigned
-1.2911	-0.2862	0.0347	0.5522	Ascomycota	Sordariomycetes	Hypocreales	Unassigned	Unassigned	Unassigned
-0.8552	-1.0947	0.2361	0.0016	Ascomycota	Sordariomycetes	Hypocreales	Unassigned	Unassigned	Unassigned
-2.0466	-1.1278	0.0031	0.0684	Ascomycota	Sordariomycetes	Microascales	Microascaceae	Scedosporium	S. boydii
-2.7430	-2.2308	0.0045	0.0121	Ascomycota	Sordariomycetes	Sordariales	Chaetomiaceae	Chaetomium	Unassigned
-0.5776	-1.0872	0.0260	0.1231	Ascomycota	Sordariomycetes	Sordariales	Unassigned	Unassigned	Unassigned
-3.8711	-2.1682	0.0000	0.0024	Ascomycota	Sordariomycetes	Unassigned	Unassigned	Unassigned	Unassigned
-2.3019	-1.1858	0.0209	0.0828	Ascomycota	Sordariomycetes	Unassigned	Unassigned	Unassigned	Unassigned
-0.6311	-1.6113	0.5153	0.0177	Ascomycota	Sordariomycetes	Unassigned	Unassigned	Unassigned	Unassigned
-1.3737	-1.4443	0.0390	0.0005	Basidiomycota	Agaricomycetes	Agaricales	Entolomataceae	Entoloma	Unassigned
-1.6992	-1.6459	0.0368	0.0040	Basidiomycota	Agaricomycetes	Trechisporales	Unassigned	Unassigned	Unassigned
-1.5827	-0.6285	0.0259	0.2748	Basidiomycota	Agaricomycetes	Unassigned	Unassigned	Unassigned	Unassigned

-1.0304	-0.8712	0.0435	0.2622	Basidiomycota	Agaricomycetes	Unassigned	Unassigned	Unassigned	Unassigned
-1.4138	-0.6591	0.0453	0.3645	Basidiomycota	Microbotryomycetes	Sporidiobolales	Unassigned	Unassigned	Unassigned
-1.2271	-1.3856	0.1364	0.0411	Basidiomycota	Microbotryomycetes	Sporidiobolales	Unassigned	Unassigned	Unassigned
-2.6642	-2.1359	0.0117	0.0208	Basidiomycota	Tremellomycetes	Tremellales	Trimorphomycetaceae	Saitozyma	S. podzolica
-2.1462	-1.1505	0.0376	0.2751	Basidiomycota	Tremellomycetes	Tremellales	Unassigned	Unassigned	Unassigned
-2.8139	-2.3758	0.0030	0.0140	Chytridiomycota	Rhizophydiomycetes	Rhizophydiales	Rhizophydiaceae	Rhizophydium	Unassigned
-3.4823	-2.9304	0.0040	0.0134	Mortierellomycota	Mortierellomycetes	Mortierellales	Mortierellaceae	Mortierella	Unassigned
2.0376	1.6755	0.0247	0.0034	Ascomycota	Dothideomycetes	Venturiales	Sympoventuriaceae	Veronaeopsis	Unassigned
1.9648	1.9877	0.0170	0.0048	Ascomycota	Leotiomycetes	Helotiales	Xylogone	Unassigned	Unassigned
2.3568	1.1905	0.0035	0.0134	Ascomycota	Sordariomycetes	Glomerellales	Plectosphaerellaceae	Verticillium	V. leptobactrum
2.8294	2.1790	0.0095	0.0033	Ascomycota	Sordariomycetes	Hypocreales	Clavicipitaceae	Metarhizium	Unassigned
1.8250	1.8560	0.0227	0.0048	Ascomycota	Sordariomycetes	Hypocreales	Nectriaceae	Unassigned	Unassigned
0.1720	0.5774	0.3978	0.0336	Ascomycota	Sordariomycetes	Xylariales	Xylariaceae	Xylaria	Unassigned
0.7148	-0.0085	0.0458	0.9906	Basidiomycota	Agaricomycetes	Agaricales	Tricholomataceae	Tricholoma	T. matsutake
2.7337	2.7013	0.0130	0.0022	Basidiomycota	Agaricomycetes	Polyporales	Ganodermataceae	Ganoderma	G. australe
2.1326	2.1449	0.0221	0.0035	Basidiomycota	Agaricomycetes	Polyporales	Ganodermataceae	Ganoderma	G. multiplicatum
2.2171	2.4152	0.0137	0.0020	Basidiomycota	Agaricomycetes	Polyporales	Meripilaceae	Rigidoporus	Unassigned
1.2410	1.4567	0.0718	0.0068	Basidiomycota	Dacrymycetes	Dacrymycetales	Dacrymycetaceae	Dacryopinax	Unassigned
1.8457	1.4395	0.0286	0.0108	Basidiomycota	Tremellomycetes	Tremellales	Unassigned	Unassigned	Unassigned
2.0219	1.2651	0.0156	0.0238	Basidiomycota	Tremellomycetes	Trichosporonales	Trichosporonaceae	Apiotrichum	A. scarabaeorum
1.5780	0.8322	0.0425	0.0484	Chytridiomycota	Rhizophydiomycetes	Rhizophydiales	Rhizophydiaceae	Rhizophydium	Unassigned
1.5378	1.5299	0.0252	0.0262	Mortierellomycota	Mortierellomycetes	Mortierellales	Mortierellaceae	Mortierella	Unassigned
0.5007	1.8636	0.1434	0.0125	Mortierellomycota	Mortierellomycetes	Mortierellales	Unassigned	Unassigned	Unassigned
1.5715	2.1343	0.0419	0.0061	Mortierellomycota	Mortierellomycetes	Mortierellales	Unassigned	Unassigned	Unassigned
