# Supplementary material for: Leafcutter ants enhance microbial drought resilience in tropical forest soil
Source: Environ Microbiol Rep. 2024 May 23;16(3):e13251. doi: 10.1111/1758-2229.13251 (PMC11112399; doi:10.1111/1758-2229.13251)
Supplement: Supplementary file 2 — Data S1: Supporting Information. [file EMI4-16-e13251-s002.docx]

**Experimental Procedures**

*Study Site:* This study was conducted at La Selva Biological Station, an old growth tropical wet forest reserve in Cordillera Central, Costa Rica. We have previously described climatic and methane flux patterns at this site [(Aronson *et al.*, 2019)](https://paperpile.com/c/Vlebbn/KAXx). The mean monthly rainfall at La Selva is typically above 300 mm from May to December with precipitation peaking above 400 mm/month in June–August and November– December and with the driest period in February-March receiving above 150 mm [(Organization for Tropical Studies)](https://paperpile.com/c/Vlebbn/ch2ko). Due to an El Nino event occurring during this study in 2016, soils were exceptionally dry in April and May The dry conditions peaked on May 8th, when the water content was 27% and the water potential approximately -5MPa, well below the permanent wilting point (-1.5MPa) of 33% [(Sollins *et al.*, 1994)](https://paperpile.com/c/Vlebbn/xVGAh).

*Sample Collection***:** In both residual and soil regions**,** 2 plots were set up in areas both occupied by *Atta cephalotes* ant nests and in non-nest control soils (sFig. 1). Each plot was divided into 4 even subplots (n=4), and collars were set up near plot corners, 1 in each of the 4 subplots. Soil samples were collected from the top 5cm of each subplots 4 times during the study period: March, May, July, and September of 2016. After collecting, soils were stored at -20C. After the final collection in September, DNA was extracted from all soils using the MOBIO PowerLyzer Powersoil kit (MOBIO Laboratories Inc, Carlsbad CA, Catalog # 12855-100) according to the manufacturer’s instructions.

Soil CH_4_ flux was collected from the collars using a closed-chamber system described previously [(Aronson *et al.*, 2019)](https://paperpile.com/c/Vlebbn/KAXx). Briefly, collars were sealed with PVC caps and left to incubate for 40 minutes. Chamber air sampled at three timepoints was analyzed on a gas chromatograph (7890B, Agilent Technologies, Santa Clara, California, USA) to determine CH_4_ flux. Volumetric water content and soil temperature in the top 5cm were also collected next to each collar following the flux measurement using a ProCheck handheld datalogger with GS3 sensor (Decagon Devices, Pullman, WA, USA).

*Marker Gene Amplicon Sequencing:* To target bacterial communities, the V3-V4 region of the 16S rRNA gene was amplified from soil DNA extracts using the S-D-Bact-0341-b-S-17 (​​5'-CCTACGGGNGGCWGCAG-3') and S-D-Bact-0785-a-A-21 (5'-GACTACHVGGGTATCTAATCC-3') primer set [(Klindworth *et al.*, 2013)](https://paperpile.com/c/Vlebbn/WcGxX). DNA was amplified using KAPA HiFi HotStart ReadyMix (Roche Diagnostics, Indianapolis, IN, United States) and 0.2 μM of each primer. The reaction was carried out with the following thermocycle: Initial denaturing at 95°C for 3 minutes, followed by 25 cycles of 95°C for 30 seconds, 55°C for 30 seconds, 72°C for 30 seconds, and concluding with a final extension at 72°C for 5 minutes.

To target fungal communities, the internal transcribed spacer 2 (ITS2) region was amplified from soil DNA extracts using the 5.8S-F (5'-AACTTTYRRCAAYGGATCWCT-3') /ITS4-FunR (5'-AGCCTCCGCTTATTGATATGCTTAART-3') primer set [(Taylor *et al.*, 2016)](https://paperpile.com/c/Vlebbn/5HuCy). DNA was amplified using the Phusion High Fidelity Master Mix (NEB), an additional 3mM MgCl_2_, and 0.2 μM of each primer. The reaction was carried out with the following thermocycle: Initial denaturing at 95°C for 2 minutes, followed by 35 cycles of 95°C for 30 seconds, 55°C for 30 seconds, and 60°C for 4 minutes. Libraries were indexed and then sequenced with paired ended 300bp reads on the Illumina MiSeq platform. The 16S library was sequenced across two MiSeq runs and the ITS2 library was sequenced in a single MiSeq run. These sequence data have been submitted to the SRA under accession number PRJNA749330.

*Bioinformatics*: Demultiplexed 16S sequences were analyzed in QIIME2 [(Bolyen *et al.*, 2019)](https://paperpile.com/c/Vlebbn/mYehJ). To minimize batch effect due to sequencing runs, only forward reads were used to analyze bacterial communities. Reads were trimmed to 230 base pairs in QIIME2. DADA2 was then used to remove chimeras, quality filter, and sort reads into amplicon sequence variants (ASVs). 16S sequences were assigned taxonomy with a bayesian classifier using reference sequences from the SILVA database release 132 [(Quast *et al.*, 2013)](https://paperpile.com/c/Vlebbn/YMfVU). ITS2 reads were processed using the amptk bioinformatics pipeline [(Palmer *et al.*, 2018)](https://paperpile.com/c/Vlebbn/kNrWb). Briefly, USEARCH9 was used to merge paired-end reads, cluster sequences, and pick ASVs. ASVs were assigned taxonomy using the amptk custom ITS2 database and hybrid taxonomy assignment algorithm. For both 16S and ITS2 datasets, data from a negative sequencing control was used to remove <20 contaminant ASVs. Due to low read counts or rarefactions curves indicating poor library amplification, 6 bacterial samples and 5 fungal samples were removed. After processing and filtering, there were a total of 6,363,877 bacterial and 3,622,525 fungal reads classified into 19,142 bacterial and 13,206 fungal ASVs. After filtering out all ASVs whose cumulative read depth accounted for less than 0.05%, there were 1,698 bacterial ASVs and 1,709 fungal ASVs.

*Microbial Community Analysis*: Community composition was analyzed by Principal Coordinate Analysis of an aitchison distance matrix using the Phyloseq package in R [(McMurdie and Holmes, 2013)](https://paperpile.com/c/Vlebbn/opyD5) using ASV counts normalized using centered log-ratio (clr) transformation in the R package ANCOMBC [(Lin and Peddada, 2020)](https://paperpile.com/c/Vlebbn/mgW3f). Log fold changes of common ASVs from March to May were calculated using ANCOMBC. Alpha diversity was analyzed by calculating species richness, evenness, and the ratio of common to rare species richness.

*Methanotroph gene abundance:* To determine the abundance of methanotrophic bacteria, we performed quantitative PCR on the *pmoA^^[[1]](#footnote-1)^^* gene. Reactions were performed in triplicate using the Biorad C1000 thermocycler. The *pmoA* gene was amplified with the A189F (5’-GGNGACTGGGACTTCTGG-3’)/Mb661R (5’-CCGGMGCAACGTCYTTACC-3’) primer set [(Kolb *et al.*, 2003)](https://paperpile.com/c/Vlebbn/K1rVL) at a final concentration of 0.25 μM, 1 μL template, and 1X Forget-Me-Not EvaGreen qPCR mastermix. The final PCR mixture also included 5% PEG, 2.5 mM MgCl_2_, and 250 μg/μL BSA. The PCR reaction was first heated to 37°C for 20 min to denature contaminants and then initial template denaturation was performed at 95°C for 2 minutes. This was followed by 40 cycles of 95°C for 30 seconds, 57°C for 45 seconds, 72°C for 30 seconds, and a final 59°C capture step. The capture step temperature was determined by testing the PCR reaction with a 50-95°C melt curve and used to exclude nonspecific amplification from quantification. A standard curve using the 759 bp *Methylocystis* *pmoA* BN69_2927 gene was used to calculate the total copies of *pmoA* and standardized to soil weight and moisture. Efficiency of qPCR reactions was at least 84% with R^2^ =0.999.

*Statistical analysis****:*** Three-way repeated measures ANOVA models were used to determine the impact of the 3 experimental factors (soil type, ant nests, and sampling month) on soil moisture, CH_4_ fluxes, alpha diversity, the ratio of common:rare taxa richness, and microbial gene abundance and expression data. Post hoc analyses included 2-way repeated measure ANOVAs to analyze the impacts of nests and sampling month within each soil group and Tukey tests to compare month to month means within each soil and nest group.

PERMANOVAs were performed on the aitchison distance matrices in order to determine the impact of these 3 experimental factors on microbial composition, as well as the relationship between microbial composition and CH_4_ flux. ANOVAs were run in base R and PERMANOVAs were run using the adonis package. Batch effect was included as a random effect in the analyses of bacterial sequencing data.

**References:**

[Aronson, E.L., Dierick, D., Botthoff, J.K., Oberbauer, S., Zelikova, T.J., Harmon, T.C., et al. (2019) ENSO‐Influenced Drought Drives Methane Flux Dynamics in a Tropical Wet Forest Soil. *J Geophys Res Biogeosci* **124**: 2267–2276.](http://paperpile.com/b/Vlebbn/KAXx)

[Bolyen, E., Rideout, J.R., Dillon, M.R., Bokulich, N.A., Abnet, C.C., Al-Ghalith, G.A., et al. (2019) Reproducible, interactive, scalable and extensible microbiome data science using QIIME 2. *Nat Biotechnol* **37**: 852–857.](http://paperpile.com/b/Vlebbn/mYehJ)

[Clark, D.B., Palmer, M.W., and Clark, D.A. (1999) Edaphic Factors and the Landscape-Scale Distributions of Tropical Rain Forest Trees. *Ecology* **80**: 2662–2675.](http://paperpile.com/b/Vlebbn/XjaMI)

[Delgado-Baquerizo, M., Eldridge, D.J., Hamonts, K., and Singh, B.K. (2019) Ant colonies promote the diversity of soil microbial communities. *ISME J* **13**: 1114–1118.](http://paperpile.com/b/Vlebbn/WSAi)

[Foster, Z.S.L., Sharpton, T.J., and Grünwald, N.J. (2017) Metacoder: An R package for visualization and manipulation of community taxonomic diversity data. *PLoS Comput Biol* **13**: e1005404.](http://paperpile.com/b/Vlebbn/zB1ZT)

[Klindworth, A., Pruesse, E., Schweer, T., Peplies, J., Quast, C., Horn, M., and Glöckner, F.O. (2013) Evaluation of general 16S ribosomal RNA gene PCR primers for classical and next-generation sequencing-based diversity studies. *Nucleic Acids Res* **41**: e1.](http://paperpile.com/b/Vlebbn/WcGxX)

[Kolb, S., Knief, C., Stubner, S., and Conrad, R. (2003) Quantitative detection of methanotrophs in soil by novel pmoA-targeted real-time PCR assays. *Appl Environ Microbiol* **69**: 2423–2429.](http://paperpile.com/b/Vlebbn/K1rVL)

[Lin, H. and Peddada, S.D. (2020) Analysis of compositions of microbiomes with bias correction. *Nat Commun* **11**: 3514.](http://paperpile.com/b/Vlebbn/mgW3f)

[Love, M.I., Huber, W., and Anders, S. (2014) Moderated estimation of fold change and dispersion for RNA-seq data with DESeq2. *Genome Biol* **15**: 550.](http://paperpile.com/b/Vlebbn/u9kb6)

[McMurdie, P.J. and Holmes, S. (2013) phyloseq: an R package for reproducible interactive analysis and graphics of microbiome census data. *PLoS One* **8**: e61217.](http://paperpile.com/b/Vlebbn/opyD5)

[Organization for Tropical Studies Meteorological data.](http://paperpile.com/b/Vlebbn/ch2ko)

[Palmer, J.M., Jusino, M.A., Banik, M.T., and Lindner, D.L. (2018) Non-biological synthetic spike-in controls and the AMPtk software pipeline improve mycobiome data. *PeerJ* **6**: e4925.](http://paperpile.com/b/Vlebbn/kNrWb)

[Quast, C., Pruesse, E., Yilmaz, P., Gerken, J., Schweer, T., Yarza, P., et al. (2013) The SILVA ribosomal RNA gene database project: improved data processing and web-based tools. *Nucleic Acids Res* **41**: D590–6.](http://paperpile.com/b/Vlebbn/YMfVU)

[Sollins, P., M., F.S., Ch., R.M., and Sanford, R.L. (1994) Soils and Soil Process Research. *Trends Ecol Evol* 34–54.](http://paperpile.com/b/Vlebbn/xVGAh)

[Taylor, D.L., Walters, W.A., Lennon, N.J., Bochicchio, J., Krohn, A., Caporaso, J.G., and Pennanen, T. (2016) Accurate Estimation of Fungal Diversity and Abundance through Improved Lineage-Specific Primers Optimized for Illumina Amplicon Sequencing. *Appl Environ Microbiol* **82**: 7217–7226.](http://paperpile.com/b/Vlebbn/5HuCy)

1. To investigate methanotroph abundance in this study, we used a primer set that targets the largest available methanotroph diversity [(Kolb *et al.*, 2003)](https://paperpile.com/c/vOY4QA/qJhzb), capturing both gammaproteobacterial type I methanotrophs and alphaproteobacterial type II methanotrophs [(Hanson and Hanson, 1996)](https://paperpile.com/c/vOY4QA/PSerh). However, this primer set has been demonstrated to have limited efficacy capturing the diversity of Verrucomicrobial methanotrophs [(Ghashghavi *et al.*, 2017)](https://paperpile.com/c/vOY4QA/5FxiV), a group that was also detected in our 16S survey data (specifically, ASVs belonging to the genus *Methylacidiphulum*). Based on this research campaign, we would recommend that investigation into methanotrophy at La Selva or similar tropical forest ecosystems take into account Verrucomicrobial methanotrophs, which may be more widespread outside extremophilic niches than originally thought [(van Teeseling *et al.*, 2014)](https://paperpile.com/c/vOY4QA/J35bT) and are capable of carrying out anaerobic methane oxidizing reactions [(Op den Camp *et al.*, 2009)](https://paperpile.com/c/vOY4QA/J3EK). [↑](#footnote-ref-1)
